# The Ketogenic Diet Does Not Affect Growth of Hedgehog Pathway Medulloblastoma in Mice

**DOI:** 10.1371/journal.pone.0133633

**Published:** 2015-07-20

**Authors:** Mai T. Dang, Suzanne Wehrli, Chi V. Dang, Tom Curran

**Affiliations:** 1 Department of Pathology and Laboratory Medicine, Children’s Hospital of Philadelphia, Philadelphia, Pennsylvania, United States of America; 2 Abramson Cancer Center, University of Pennsylvania, Philadelphia, Pennsylvania, United States of America; Indiana University School of Medicine, UNITED STATES

## Abstract

The altered metabolism of cancer cells has long been viewed as a potential target for therapeutic intervention. In particular, brain tumors often display heightened glycolysis, even in the presence of oxygen. A subset of medulloblastoma, the most prevalent malignant brain tumor in children, arises as a consequence of activating mutations in the Hedgehog (HH) pathway, which has been shown to promote aerobic glycolysis. Therefore, we hypothesized that a low carbohydrate, high fat ketogenic diet would suppress tumor growth in a genetically engineered mouse model of medulloblastoma. However, we found that the ketogenic diet did not slow the growth of spontaneous tumors or allograft flank tumors, and it did not exhibit synergy with a small molecule inhibitor of Smoothened. Serum insulin was significantly reduced in mice fed the ketogenic diet, but no alteration in PI3 kinase activity was observed. These findings indicate that while the ketogenic diet may be effective in inhibiting growth of other tumor types, it does not slow the growth of HH-medulloblastoma in mice.

## Introduction

Medulloblastoma (MB) is the most common malignant brain tumor in children accounting for approximately 25% of all pediatric brain tumors [1]. Patients with medulloblastoma are treated with a combination of surgical resection, radiation, and chemotherapy [2]. Although these therapies result in 5-year survival rates of up to 70%, they are also associated with high morbidity. Furthermore, tumors recur in 40% of treated patients with more than 30% eventually dying from the disease [3–5]. The Hedgehog (HH) pathway subtype of MB presented the first opportunity for an intervention strategy based on a molecularly-targeted therapy in this pediatric brain tumor [6–8]. Proof-of-concept studies using a Smoothened (Smo) inhibitor, demonstrated that blocking HH pathway activity inhibits tumor cell proliferation and increases apoptosis in a MB mouse model, *Ptch1* ^*+/-*^ *Trp53* ^*-/-*^, resulting in eradication of even large spontaneous brain tumors [9]. These preclinical findings were followed by promising clinical results in MB patients in early clinical trials [10, 11]. However, enthusiasm for the use of this approach has been tempered by observations of the rapid acquisition of drug resistance in all MB patients who showed initial responses [12]. Thus, there is still a great need for additional, and perhaps combinatorial, therapeutic approaches for treatment of this disease.

Many decades ago, Otto Warburg suggested that cancer cells exhibit an increased capacity to utilize glucose and produce lactate even when an adequate supply of oxygen is present [13]. While incompletely understood, increased glycolysis in tumor cells may be important for production of non-essential amino acids such as serine [14]. In cancer cells, the majority of pyruvate is converted to lactate and glutamine is utilized for replenishing tricarboxylic acid cycle intermediates [15]. In the brain, cells primarily metabolize glucose but they can switch to ketone use under low glucose conditions. The ketone bodies, acetoacetate and D-β-hydroxybutyrate, are produced in the liver via fatty acid oxidation, transported to extrahepatic tissues and taken up by cells where they enter the tricarboxylic cycle as acetyl-CoA. The brain uses ketone bodies at a rate that is directly proportional to their concentrations in arterial blood [16]. However, little is known about the utilization of ketones in brain tumor cells. Studies, carried out in mouse models, show that brain tumor growth can be reduced by placing mice on a calorie-restricted, low carbohydrate, high fat diet. Mice bearing malignant astrocytomas and orthotopically implanted human malignant gliomas exhibited reductions of 65% and 35% in tumor burden, respectively when fed a low carbohydrate, high fat diet [17]. Additionally, an orthotopic glioma mouse model showed prolonged survival after radiation treatment when maintained on a low carbohydrate, high fat diet [18]. These findings led to the proposal that a ketone-producing, low glucose diet hinders growth of brain tumor cells because they cannot efficiently use ketones when the supply of glucose is limited.

Several lines of evidence suggest that the HH subtype of MB may be particularly sensitive to the manipulation of tumor cell metabolism. The expression of key glycolytic enzymes is elevated in the *NeuroD2-SmoA1* mouse medulloblastoma model [19] and activation of the HH pathway, by treating cultured adipocytes with an agonist of SMO, induces aerobic glycolysis via AmpK and pyruvate kinase regulation [20]. Loss of the HH pathway target hexokinase 2 results in disruption of cerebellar development and reduced tumor growth in the SmoA2 mouse medulloblastoma model [21]. In addition, c-Myc, which is expressed in MB, has been shown to affect metabolism by transactivation of lactate dehydrogenase-A and suppression of microRNAs that augment mitochondrial glutaminase expression [22, 23]. Finally, insulin-like-growth factor-2 (IGF-2) is required for formation of HH-pathway tumors in mice [24], and it is increased in desmoplastic MB [25]. Here, we use a genetic mouse model of HH-MB, *Ptch1* ^*+/-*^ *Trp53* ^*-/-*^, to test the effect of a ketogenic diet on tumor growth. In addition, we investigate whether the diet can work in synergy with a SMO inhibitor to cause tumor regression. Since a low carbohydrate diet can decrease blood insulin level, we hypothesized that the diet may inhibit the PI3K pathway to slow down tumor growth.

## Material and Methods

### Animals, General Housing Conditions, and Diet

All animal housing, procedures, and euthanasia protocols were carried out in accordance with the recommendations in the Guide for the Care and Use of Laboratory Animals of the National Institutes of Health. The protocol was approved by the Committee on the Ethics of Animal Experiments of the Children’s Hospital of Philadelphia. Adult mice were euthanized by decapitation with anesthesia. P7 mice were decapitated without anesthesia.

Genetically engineered mice that develop spontaneous MB (*Ptch1* ^*+/-*^ *Trp53* ^*-/-*^ *)* were generated as described previously [26] and maintained on a mixed C57Bl/6:129SV background. Mice were group-housed (21°C; 12h:12h light:dark cycle) and given *ad libitum* access to standard rodent diet (LabDiet 5LG4) until the initiation of experiment when some groups of mice were switched to the ketogenic diet. Tumor-bearing mice were observed daily and were euthanized when they lost >20% of their body weight or showed signs of ataxia, significant decrease in activity, or hydrocephalus.

NOD SCID mice were used for allograft transplantation of spontaneous MB from *Ptch1* ^*+/-*^ *Trp53* ^*-/-*^ mice. A tumor-bearing cerebellum was resected, mechanically dissociated to near single-cell suspension, and mixed with Matrigel Basement Membrane Matrix (BD). Animals were anesthetized with isoflurane and injected subcutaneously with cell suspensions in the flank. Flank tumor width, length, and depth were measured with a digital caliper (VWR). The formula for volume calculation was volume = π/6 x average (width, length, and depth)^3^.

Two irradiated ketogenic diets with different ratios of calories from fat and protein to calories from carbohydrates were used: 4:1 (Teklad, TD.96355) and 6:1 (Bio-Serv, S3666). The paste was contained in a 6 cm plastic petri dish and replaced daily. The dish was placed in the center of the cage for *ad libitum* feeding.

### Glucose, Ketone, and Insulin Measurements

Glucose and ketone levels were measured from whole blood obtained by mandibular venous puncture carried out in the early evening between 4 to 6pm. The measurements were made with a Precision Xtra Ketone meter (Abbott) using test strips for glucose and ketones (Abbott) at day 0 and subsequently at days 2 and 7. Serum insulin was measured using the ultra-sensitive mouse insulin ELISA kit (Crystal Chem) according to the manufacturer’s instructions.

### Magnetic Resonance Imaging and Analysis of Spontaneous Tumor Volume

MRI images were obtained with a Bruker 7 Tesla ClinScan animal scanner running the Siemens Syngo acquisition software. The linear body coil was used as transmit coil and a mouse brain 2x2 array surface coil as receive coil. During the scan the mouse laid in a prone position with the surface coil positioned on top of the head. Circulating hot water in the animal bed allowed for the body temperature to be maintained at 37°C. Mice were anesthetized throughout the experiment by a gaseous mixture of isoflurane (1.5% to 2%) and oxygen. The respiration rate was continuously measured using the Small Animal Monitoring and Gating System (SA Instrument). After obtaining a series of localizers, contiguous image slices capturing the entire brain volume were obtained in the axial, sagittal and coronal directions using a turbo spin-echo sequence with a turbo factor of 7. Eighteen to 24 slices of 0.5 mm thickness were acquired interleaved with a FOV of 21 x 25 mm, an in plane resolution of 98 x 98 μm^2^ and with fat saturation. Other conditions were as follow: TR 3.650 s, TE_eff_ 55 ms (echo spacing 13.7 ms), BW 130 Hz/pixel, refocusing flip angle 180°, 2 NEX. The time of acquisition was approximately 7 to 8 minutes.

Volumetric analysis was carried out using the Vitrea Enterprise Suite software from Vital Image. The tumors were outlined in coronal planes with a computer-assisted, free-outline technique and the tumor volume was calculated by the Vitrea software.

### Western Analysis

For hexokinase measurements, tissues from cerebellum of P7 wild-type mice, adult *Ptch1* ^*+/+*^ *Trp53* ^*-/-*^, and adult *Ptch1* ^*+/-*^ *Trp53* ^*-/-*^ mice were homogenized in 1% NP-40 lysis buffer containing 50 mM Tris-HCl (pH 7.5), 150 mM NaCl, 5mM EDTA, 25 nM NaF, 2 mM Na3VO4, 0.1% sodium deoxycholate, protease inhibitor cocktail complete mini (Roche) and phosphatase inhibitor cocktail PhosSTOP (Roche). The protein content of the lysate was quantified using the Bio-Rad protein assay kit (Bio-Rad). Protein was run on a gradient SDS-PAGE gel and transferred to nitrocellulose membrane (Invitrogen). Membranes were individually incubated with hexokinase 2 (Cell Signaling, C64G5) or vincullin antibody (Sigma, V9131) overnight at 4°C and subsequently with anti-rabbit and anti-mouse horseradish peroxidase (HRP) conjugated antibodies respectively (GE Healthcare NA9340V, NA931V) for one hour at room temperature. Membranes were then incubated with chemiluminescent HRP substrate (Millipore, WBKLS0500) and exposed to x-ray film. Images were scanned and band intensities were quantified by ImageJ (NIH).

AKT and pAKT measurements was carried out using the Odyssey infrared imaging system (Li-Cor Biosciences). Lysates of flank tumors from mice treated with standard and ketogenic diets were prepared as described above. Membranes were incubated with one of the following primary antibodies in Odyssey blocking buffer overnight at 4°C: AKT (Cell Signaling, 11E7), pAKT (Cell Signaling, 110B7E), or GAPDH (Cell Signaling, 14C10). Membranes were then incubated with IRDye secondary antibodies, IRDye 680 goat anti-mouse or IRDye 800CW goat anti-rabbit antibody (Li-Cor Biosciences), for one hour at room temperature. Infrared images were taken and quantified using the Odyssey infrared imaging system.

### Immunohistochemistry

Tumor tissues from flank tumors and whole brain tissues from P7, adult *Ptch1* ^*+/+*^ *Trp53* ^*-/-*^, and adult *Ptch1* ^*+/-*^ *Trp53* ^*-/-*^ mice were fixed in 4% formaldehyde overnight and standard paraffinization was performed. Sections were cut to 5 um thickness. Sections were rehydrated in xylene and serial ethanol concentrations. Antigen retrieval was achieved with sodium citrate at sub-boiling temperature in the microwave for 30 minutes. Sections were incubated overnight at 4°C with Foxo3a antibody (Cell Signaling, D19A7) or hexokinase 2 antibody and subsequently with anti-rabbit biotinylated secondary antibody (Vector Labs, BA-1000) for 30 minutes at room temperature. For hexokinase 2, sections were incubated with avidin/biotin ABC complex (Vector Labs, PK-6102) and stained with DAB substrate chromogen (DAKO, 2016–10). For Foxo3a, after secondary antibody incubation, sections were incubated with fluorescein-labeled avidin D for 30 minutes (Vector Labs). Slides were then mounted with Vectashield DAPI mounting medium for immunofluorescence (Vector Labs). High magnification images of sections were captured using a Nikon 90i microscope equipped with Roper EZ monochrome and DS-Fi1 color cameras. NIS Elements BR 3.0 software was used to capture and analyze the images.

### Hedgehog Antagonist GDC-0449 Oral Administration

Mice with flank allografts were paired and segregated into two groups when tumors were between 75 and 100mm^3^. One group was maintained on a standard diet and the other was started on the 6:2 Bio-Serv ketogenic diet. When tumors were between 300 to 400 mm^3^, drug treatment was started. Mice were treated with the SMO inhibitor GDC-0449 at a dose of 20 mg/kg, dissolved in methylcellulose, by oral gavage twice daily for 5 days. Measurements of tumor volume were made and calculated once daily.

### Statistical Analysis

Statistical analyses of data were carried out using the unpaired two-tailed Student’s t-test for comparison between two experimental groups. Survival analysis was carried out using the log-rank test for equality of survivor functions. Differences were considered to be significant when probability (*p*) values were <0.05.

## Results

### *Ptch1* ^*+/-*^ *Trp53* ^*-/-*^ Medulloblastoma Have Heightened Glycolysis

**Fig 1:**
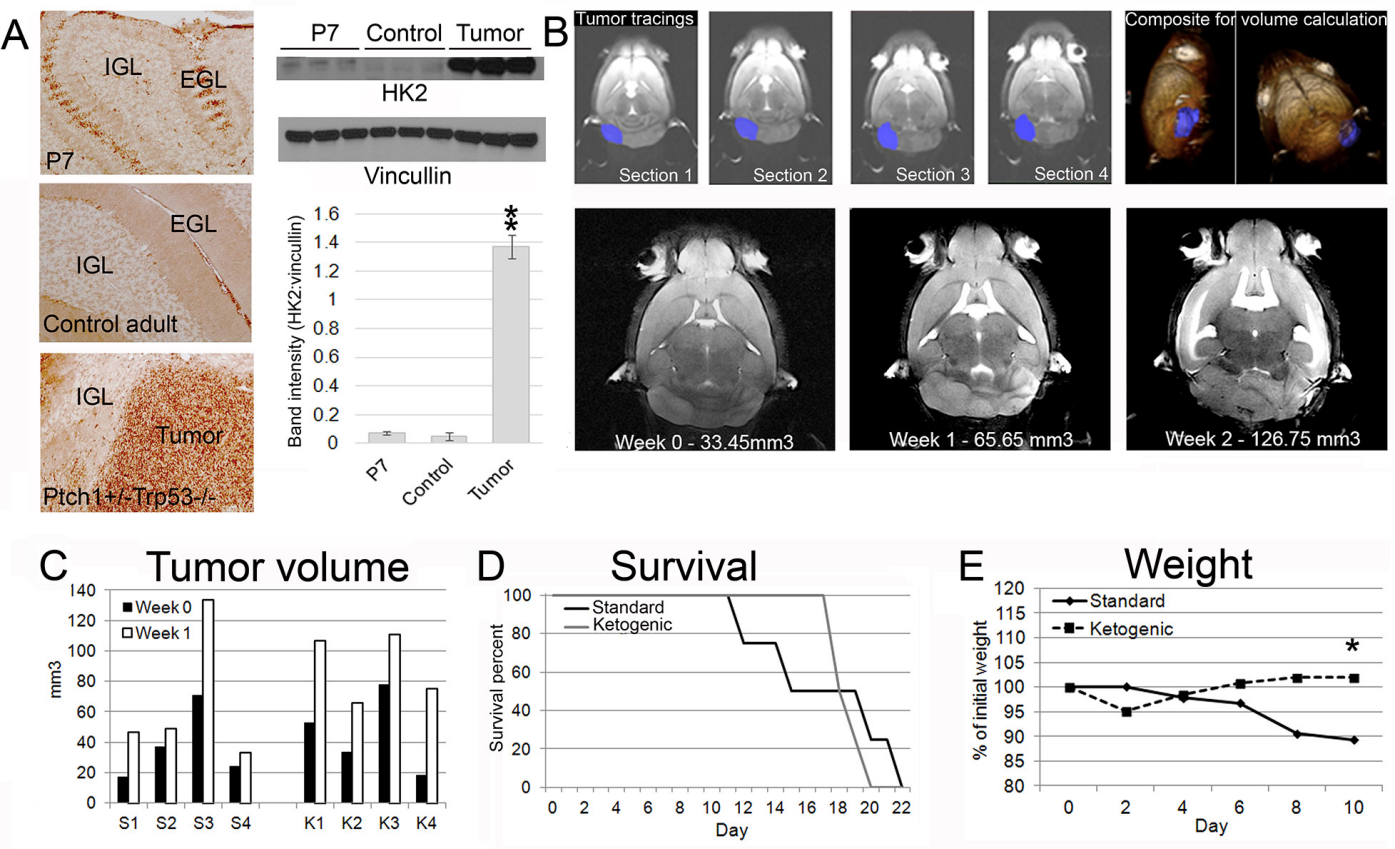
Medulloblastoma have increased HK2, and the ketogenic diet does not slow growth of *Ptch1* ^*+/-*^ *Trp53* ^*-/-*^ medulloblastoma. (A) Immunohistochemistry with HK2 antibody show tumors have increased HK2 level in comparison to normal cerebellum and comparable to a heightened level seen in the external granule layer of P7 mice containing rapidly dividing cerebellar granule neuron precursor cells. Western analysis quantification show tumor tissues have increased HK2 level over normal cerebellar tissue (*p* = 0.000012) and to whole P7 cerebellar tissue (*p* = 0.000012). EGL = external granule layer, IGL = internal granule layer. (B) Six-week old *Ptch1* ^***+/-***^ *Trp53* ^***-/-***^ mice were imaged with MRI and tumor volumes were calculated. Four consecutive MRI images of one spontaneous medulloblastoma, traced for volume calculation. Representative images of a single tumor over two weeks. (C) No changes were seen in individual tumor sizes before and after one week of ketogenic diet initiation compared to mice on a standard diet. The average percentage of tumor volume change from initial volumes in the standard and ketogenic diet groups were 182 ± 66% and 240 ± 123% respectively (*p* = 0.437). (D) The ketogenic diet did not affect survival (*p* = 0.778). (E) Mice on the standard diet lost an average of 10% of their body weight while mice on the ketogenic diet maintained their body weight (*p* = 0.030 on day 10). S = standard diet, K = ketogenic diet.

To confirm that *Ptch1* ^*+/-*^ *Trp53* ^*-/-*^ medulloblastoma have increased glycolysis, three P7 cerebella, three adult *Ptch1* ^*+/+*^ *Trp53* ^*-/-*^ cerebella and three adult *Ptch1* ^*+/-*^ *Trp53* ^*-/-*^ cerebella with tumors were analyzed for levels of hexokinase 2, the first rate-limiting enzyme in glycolysis by both immunohistochemistry and Western blot analysis. Similar to previously published reports in another HH-MB model [21], tumors have increased hexokinase 2 staining in comparison to normal cerebellum. A heightened level of HK2 staining was seen in the external granule layer of P7 mice containing rapidly dividing cerebellar granule neuron precursor cells (Fig 1A). Western blot analysis shows that HK2 level is significantly higher in cerebella with tumor in comparison to P7 whole cerebellum (*p* = 0.000012) and adult whole cerebellum (*p* = 0.000012).

### The Ketogenic Diet Does Not Affect Growth of *Ptch1* ^*+/-*^ *Trp53* ^*-/-*^ Medulloblastoma

To investigate whether the ketogenic diet can affect spontaneous tumor growth in a genetically engineered mouse model of HH-MB, eight six-week old *Ptch1* ^*+/-*^ *Trp53* ^*-/-*^ mice were imaged with MRI and tumor volumes were calculated (Fig 1B). The initial average tumor size for each group (n = 4,4) was 37 ± 24 mm^3^ (standard mouse chow) and 45 ± 26 mm^3^ (ketogenic diet, Fig 1C). One week after the diet change, mice were re-imaged and tumor volumes were calculated. The average percentage tumor growth in the standard and ketogenic diet groups were 182 ± 66% and 240 ± 123% respectively (*p* = 0.437). The ketogenic diet did not affect survival with animals from both groups requiring euthanization starting at day 10 (*p* = 0.778, Fig 1D). Mice on the standard diet lost an average of 10% of their body weight at day 10 of the experiment while mice on the ketogenic paste maintained their body weight (*p* = 0.030, Fig 1E).

### The Ketogenic Diet Does Not Affect Tumor Growth of Flank Allograft Tumors and It Does Not Accelerate Regression Induced by GDC-0449

**Fig 2:**
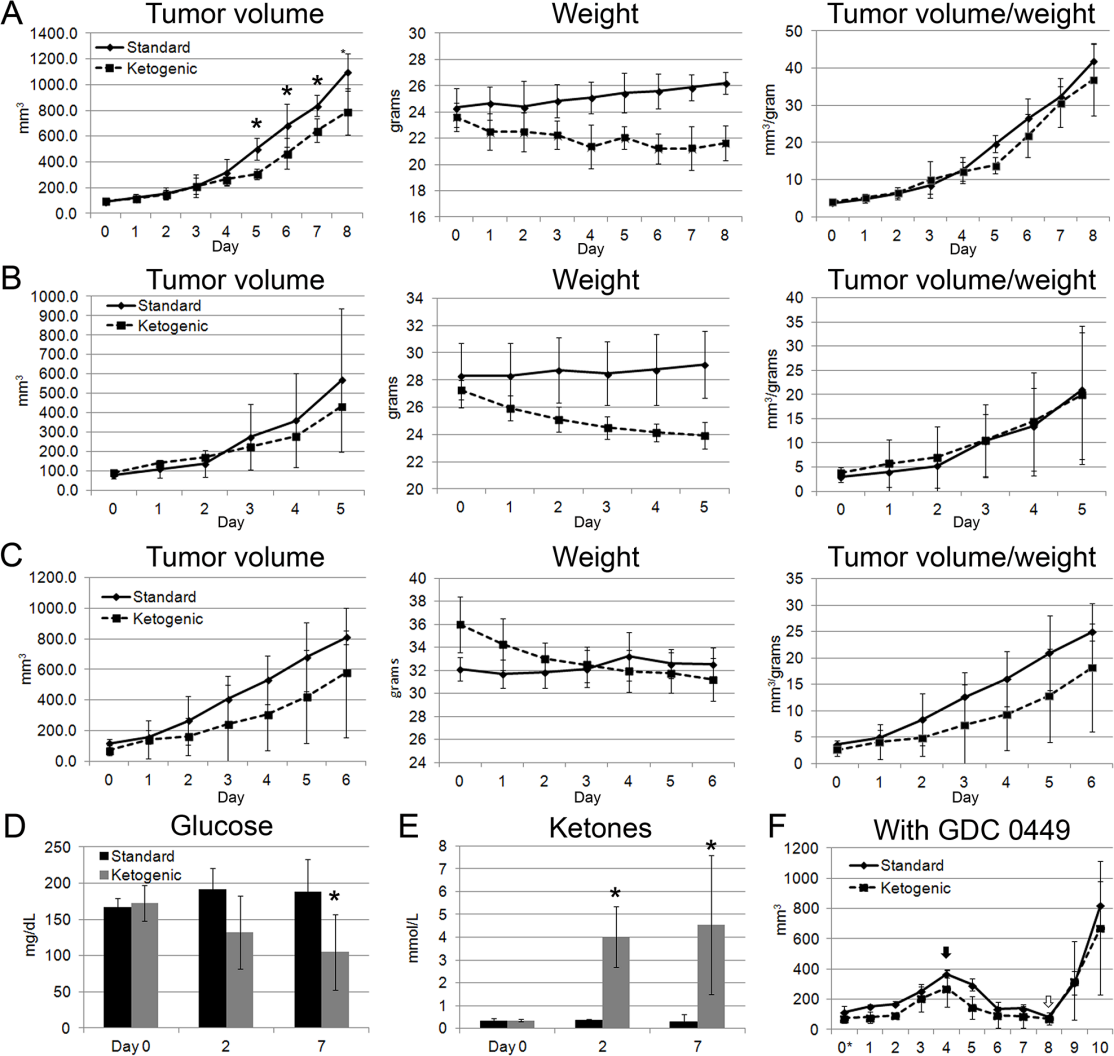
The ketogenic diet does not slow growth of flank allograft tumors, and it does not affect tumor regression induced by GDC-0449. (A-C) Three distinct spontaneous medulloblastoma from *Ptch1* ^***+/-***^ *Trp53* ^***-/-***^ mice were used for these studies. Tumor volume differences were not statistically significant in two of the three trials. In the first trial, tumor volume was significantly lower at several time points (*p* = 0.001 to 0.048 on days 5 to 8; Student t-test). However, the difference was not evident when tumor volume was calculated as a percentage of total animal weight. Mice in all three trials lost approximately 9 to 13% of their body weight on the ketogenic diet whereas those on the standard diet maintained or marginally gained weight. (D) Glucose levels were significantly lower by day 7 (*p* = 0.027). (E) Ketone levels were significantly different between the standard and ketogenic diet groups (*p* = 0.0003 at 2 days; *p* = 0.013 at 7 days). (F) The ketogenic diet did not affect tumor regression induced by the SMO inhibitor GDC-0449. Tumor volumes regressed similarly on inhibitor treatment and had similar regrowth when removed from treatment.

Next, we confirmed the results in an allograft model that allowed daily tumor volume measurements. For this study, we used a higher ratio ketogenic paste diet of 6:1. Three separate spontaneous MB from *Ptch1* ^*+/-*^ *Trp53* ^*-/-*^ mice were subcutaneously injected into the flanks of three groups of NOD SCID mice. Tumors were measurable between 3 to 5 days post-injection. Mice with tumors between 75mm^3^ to 100mm^3^ were paired; one was assigned to continue receiving the standard rodent pellet chow and the other to the 6:1 ketogenic paste. Each pair was euthanized when either mouse had a tumor >1000mm^3^. All mice appeared to have normal activity levels by observation and did not demonstrate signs of distress from tumor burden throughout the experiment. The ketogenic diet did not inhibit allograft tumor growth. In the first trial, the volume was significantly different at several time points (n = 5,5; *p* = 0.001 to 0.048 on days 5 to 8; Student t-test, Fig 2A–2C) but the difference was eliminated when tumor volume per mouse weight was analyzed. Mice in all three trials lost approximately 9 to 13% of their body weight on the ketogenic diet and those on the standard diet maintained or marginally gained weight.

The ketogenic diet led to a significantly lower glucose level by day 7, with values ranging from 160 to 237mg/dL in mice on a standard diet versus 60 to 180mg/dL in mice on the ketogenic diet (n = 5,5; *p* = 0.027, Student’s t-test, Fig 2D). Ketone levels significantly increased within 2 days of diet change, reaching an average of 4 mmol/L (n = 5,5; *p* = 0.0003 at 2 days, *p* = 0.013 at 7 days, Fig 2E).

The ketogenic diet did not affect GDC-0449-induced regression of tumor volume. In both groups, tumors regressed to less than 30% of their original volume after 5 days of treatment regardless of the diet (n = 3,3; Fig 2F). No difference was observed in tumor regrowth in both groups after 2 days off therapy.

### The Ketogenic Diet Decreases Insulin but Does Not Affect the PI3K Pathway

**Fig 3:**
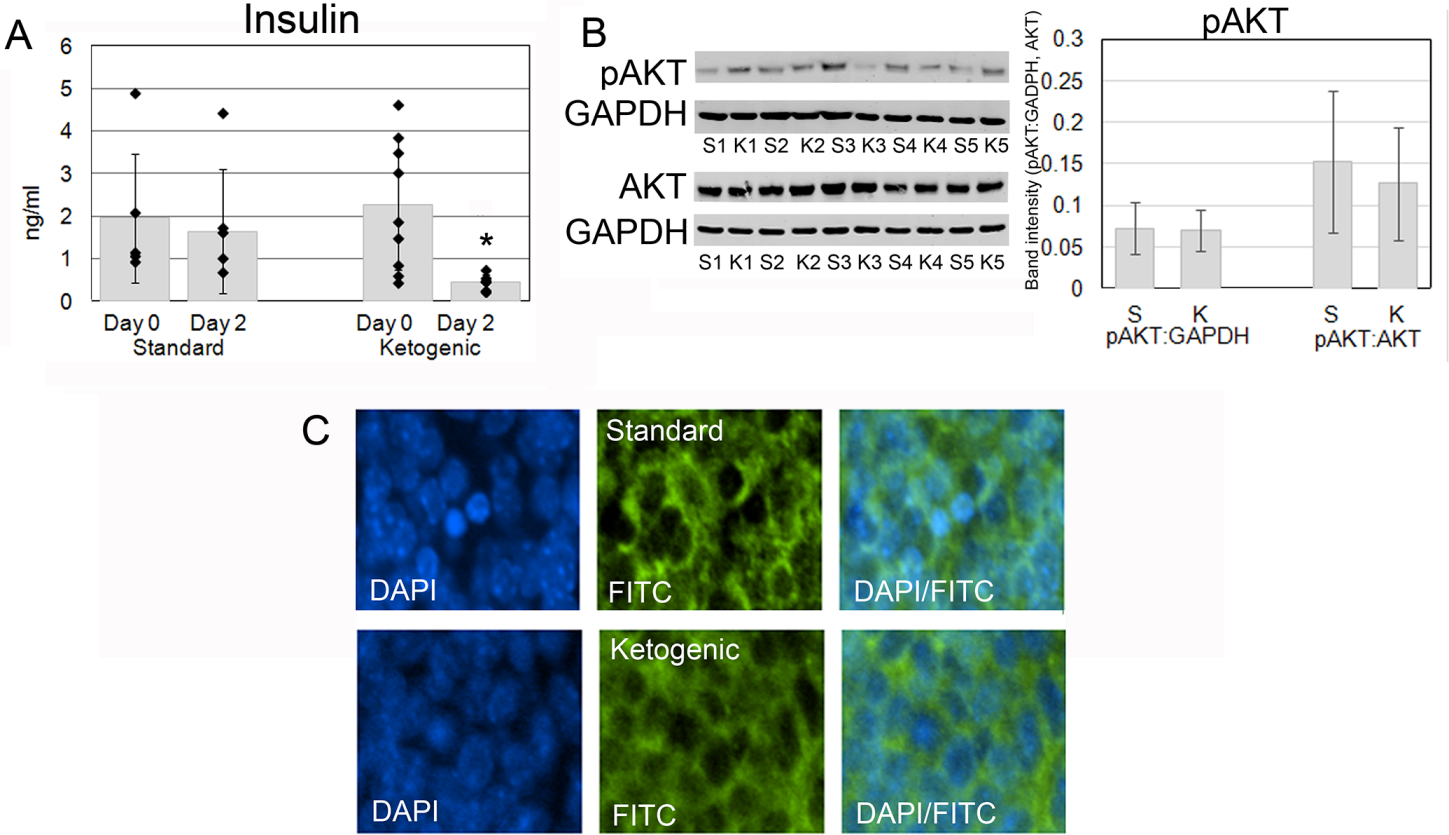
The ketogenic diet causes a significant decrease in insulin, but does not affect the PI3K pathway. (A) Insulin levels were significantly reduced by 2 days after initiation of the diet. Average serum concentration was 1.64 ng/ml on the standard diet in comparison to 0.45 ng/ml on the ketogenic diet (*p* = 0.003). (B) Phosphorylated AKT was present in tumor tissues and the level of phosphorylation was not significantly different between the two groups treated with different diets (*p* = 0.89 standardized to GAPDH, *p* = 0.64 standardized to AKT). (C) Foxo3a was localized in the cytoplasm independent of the diet. S = standard diet, K = ketogenic diet.

Insulin levels were significantly reduced 2 days after initiation of the ketogenic diet. The average serum concentration was 1.64 ng/ml on the standard diet in comparison with 0.45 ng/ml on the ketogenic diet (n = 5,9; *p* = 0.003, Fig 3A). Insulin is a known regulator of PI3K pathway. Insulin and insulin-like growth factors activate PI3K which phosphorylates AKT [27]. Among the multiple functions of AKT is phosphorylation of forkhead box O (FoxO) transcription factor family proteins in the nucleus. Phosphorylation displaces the FoxO transcription factors from target genes that promote apoptosis or cell-cycle arrest and trigger their relocalization to the cytoplasm [28]. We measured the level of phosphorylated AKT and stained for localization of Foxo3a in mice with ketogenic diet-induced insulin reduction. Phosphorylated AKT was present at low levels in HH-MB tissue and was not significantly different between the two groups treated with different diets (Fig 3B, *p* = 0.89 standardized to GAPDH, *p* = 0.64 standardized to AKT). Foxo3a was mainly localized to the cytoplasm and also was unchanged in both groups (Fig 3C).

## Discussion

In this study, we tested the effect of a calorically-unrestricted ketogenic diet on the growth of HH-MB. Using both allograft and spontaneous MB mouse models we found that the ketogenic diet had no effect on tumor growth. Previous animal studies have shown that the ketogenic diet can reduce the growth of glioblastoma, gastric, pancreatic, lung, and prostate cancer xenografts [18, 29–32]. These results led to 12 clinical trials in which cancer patients are placed on a ketogenic diet [33]. In half of these cases the diet is used as adjunctive therapy together with chemotherapy or radiation for the treatment of glioblastoma. In many cases, patients seem to be adopting the diet themselves [34]. Therefore, it is important to test the efficacy of the diet in preclinical models of other tumor types, such as we have done in this study, before this strategy is adopted widely.

Consistent with other published studies, we found that serum ketone levels were significantly increased and glucose levels only mildly decreased in mice on the ketogenic diet [17, 35]. The ketogenic diet when used in conjunction with radiation has been shown to significantly reduce regrowth of orthotopic transplanted glioblastoma and flank lung tumors [18, 19]. There are only a few cases in which the ketogenic diet has been used in conjunction with drug-based therapies in mice. We did not find any effect of the diet on the rate of regression or regrowth of tumors following treatment with a SMO inhibitor.

Among the many metabolic changes induced by the ketogenic diet is the possible alteration of insulin levels. While a low carbohydrate diet does not consistently lower serum insulin and insulin-like growth factor (IGF) levels in tumor animal models [31, 36], in our mice we did see a significant reduction in insulin. The mitogenic effect of insulin and IGF is due, in part, to activation of the PI3K pathway, one of several targets of metformin which is currently being investigated as an anti-cancer drug (reviewed in [37]). In a study of prostate cancer xenografts, the ketogenic diet was shown to cause lowered insulin and IGF, decreased phosphorylation of AKT, and slowed tumor growth [38]. However, tumor cells harboring activating mutations in PI3K were found to be resistant to caloric restriction, despite the reduction in insulin and IGF-1 levels [38]. In HH-MB, reduced insulin levels did not affect AKT phosphorylation or localization of Foxo3a [31]. Our findings suggest that insulin reduction by the ketogenic diet does not predict PI3K pathway inhibition.

The effect of a low or no carbohydrate diet on weight is variable depending on the protein and fat content as well as the caloric allowance [39, 40]. In our study, NOD SCID mice with allograft tumors exhibited weight loss when placed on the ketogenic diet. However, this was not seen in the spontaneous tumor model. *Ptch1* ^*+/-*^ *Trp53* ^*-/-*^ mice fed on the ketogenic diet maintained their body weight much longer than mice fed the standard diet. Previously, it was suggested that a low carbohydrate diet can preserve protein levels under energy restrictive conditions [41]. NOD SCID mice, even with large flank tumors, showed normal activity with no signs of stress throughout the course of the experiment. In contrast, *Ptch1* ^*+/-*^ *Trp53* ^*-/-*^ mice with spontaneous tumors in the cerebellum may experience stress as the tumor grows which could result in energy restriction. In pancreatic and colon cancer mouse models, the ketogenic diet was protective against cachexia, a tumor-induced progressive atrophy of adipose tissue and skeletal muscle [30, 42, 43]. Our findings may provide further support that the diet may protect against cachexic weight loss.

We show in this study that a low carbohydrate, high fat diet does not impact HH- MB growth or survival in our genetically engineered mouse model and flank tumor allografts. While ketones are increased and insulin is decreased, these changes in energy substrates and signaling molecules did not influence tumor growth. The alteration in diet and energy source also did not affect the efficacy of the SMO inhibitor. In conclusion, a low-carbohydrate, high-fat diet does not cause a reduction in tumor growth of HH-MB and caution should be taken when its use is considered in human patients with HH-MB, even as adjunctive tumor treatment.
